# The basics of PET molecular imaging in neurodegenerative disorders with dementia and/or parkinsonism

**DOI:** 10.1007/s00330-025-11388-5

**Published:** 2025-02-06

**Authors:** Angela Bronte, Elena Prieto, Gemma Quincoces, Elena Erro, Javier Arbizu

**Affiliations:** 1 Department of Nuclear Medicine, Clínica Universidad de Navarra, Pamplona, Spain; 2 Department of Radiophysics, Clínica Universidad de Navarra, Instituto de investigación sanitaria de Navarra (IDISNA), Pamplona, Spain; 3 Unit of Radiopharmacy, Department of Nuclear Medicine, Clínica Universidad de Navarra, Instituto de investigación sanitaria de Navarra (IDISNA), Pamplona, Spain; 4 Department of Neurology, Hospital Universitario de Navarra, Instituto de investigación sanitaria de Navarra (IDISNA), Pamplona, Spain; 5 Present Address: Department of Nuclear Medicine, Clinica Universidad de Navarra. Instituto de investigación sanitaria de Navarra (IDISNA), Pamplona, Spain

**Keywords:** Brain diseases, Molecular imaging, Positron Emission Tomography, Single photon emission computed tomography

## Abstract

**Abstract:**

Positron emission tomography (PET) imaging biomarkers have become crucial in understanding and diagnosing neurodegenerative disorders. PET imaging allows for the in vivo quantification of molecular targets with high sensitivity, aiding in the study of disease pathophysiology and progression from preclinical stages. By visualising specific molecular pathologies, PET biomarkers enable a shift from symptom-based to biology-based definitions of neurodegenerative diseases, allowing for earlier and more accurate detection and diagnosis. This has significant implications for developing and testing new therapies aimed at modifying disease course.

In this review, we will go through the standards of PET imaging in the evaluation of neurodegenerative disorders. Specifically, we will review PET molecular imaging of amyloid-β plaques, tau pathology, as well as the effect of neurodegeneration on neuronal activity in different disorders. Moreover, we will revise PET tracers targeting neurotransmitter systems such as the dopaminergic system which can detect early functional changes in movement disorders. Issues related to methods, image interpretation, normal findings and mimics will be an important part of this review.

**Key Points:**

***Question*** *A review of PET molecular imaging tools for assisting the clinical diagnosis of patients presenting with cognitive impairment or parkinsonism and suspected neurodegenerative disease*.

***Findings*** *PET molecular imaging tools vary widely in their image acquisition protocols and image interpretation, allowing us to study different features of neurodegenerative diseases*.

***Clinical relevance*** *The majority of PET molecular imaging tools are currently in use in our clinical practice. Despite the differences between them, standardised visual reading methods and specific semi-quantitative parameters have been established, allowing for their use*.

## Introduction

The current trend in defining clinical diagnostic criteria of neurodegenerative diseases is towards a more biological approach, focusing on molecular and cellular mechanisms rather than clinical symptoms alone. In addition, neurodegenerative diseases are increasingly being characterised as proteinopathies, with specific protein aggregates serving as hallmarks for different conditions. As a result, there is an increasing focus on identifying biomarkers for early diagnosis and monitoring disease progression, particularly as new disease-modifying treatments become available.

Positron emission tomography (PET) imaging biomarkers have become crucial in understanding and diagnosing neurodegenerative disorders. PET imaging allows for the in vivo quantification of molecular targets with high sensitivity, aiding in the study of disease pathophysiology and progression from preclinical stages. By visualising specific molecular pathologies, PET biomarkers enable a shift from symptom-based to biology-based definitions of neurodegenerative diseases, allowing for earlier and more accurate detection and diagnosis. This has significant implications for developing and testing new therapies aimed at modifying disease course.

PET tracers that bind to amyloid-β plaques, particularly currently available fluorinated tracers, enable the detection of amyloid pathology years before clinical symptoms of Alzheimer’s disease (AD) appear as it is one of the main neuropathological hallmarks, although it is not specific. Amyloid PET can identify individuals in the preclinical stages of AD, allowing for earlier intervention and recruitment into clinical trials [1]. Newer tau PET tracers can visualise neurofibrillary tangles, another hallmark of AD pathology. Tau PET may be able to track disease progression and neurodegeneration more closely than amyloid imaging [2]. However, the most available tracer worldwide is FDG-PET, which measures glucose metabolism and detects subtle changes in brain function before structural changes are apparent on MRI. Characteristic patterns of hypometabolism can be seen in the early stages of various neurodegenerative diseases such as AD, frontotemporal dementia, primary progressive aphasia, progressive supranuclear palsy (PSP), and dementia with Lewy bodies [3].

Although general concepts about the usefulness of PET neuroimaging in clinical practice and research are generally well-known, the details of imaging performance and interpretation are usually not familiar to prescribers and practitioners. Consequently, the aim of this review is to revise the standards of PET imaging in neurodegenerative disorders with dementia and/or parkinsonism. In particular, we will address the practical issues related to image procedures and interpretation, including image reading (normal and disease-related patterns) and quantification. In addition, we will also examine the potential pitfalls and artefacts that may arise in this process.

This review is structured according to the PET biomarkers that form the basis of this imaging technique, targeting the different molecular pathways involved in neurodegeneration and providing an overview of the different pathological processes and complementary information. We focus on the PET radiotracers that are currently available on the market and have a role in clinical practice in the evaluation of patients with cognitive impairment and/or parkinsonism, and the parameters that these radiotracers show: Protein aggregation (amyloid and tau PET imaging), neuronal activity, and neurodegeneration (2-[18F]fluoro-2-deoxy-d-glucose [^18^F]FDG) and presynaptic dopaminergic activity (L-6-[18F]fluorodopa PET, DAT SPECT, and PET).

## Protein aggregates imaging

### Amyloid PET imaging

The extracellular deposition of β-amyloid (Aβ) plaques in the cortical grey matter of the brain is one of the pathological hallmarks of Alzheimer's disease (AD) [1]. However, in most of the non-AD dementias, Aβ plaques increase with age and APOE ε4 carrier status.

In AD, the majority of the neuritic plaques are located in the frontal cortex (particularly the orbital and medial frontal cortex), cingulate gyrus, precuneus, and lateral parietal and temporal regions. However, the presence of Aβ plaques is scarce in the primary sensorimotor and occipital cortex and the mesial temporal areas [4]. Interestingly, Aβ plaques develop many years before the onset of cognitive impairment and dementia [4, 5]. Even though the distribution and density of Aβ plaques do not correlate with the degree of cognitive impairment [4].

Amyloid PET radiotracers have been developed that bind to fibrillar Aβ plaques in the brain, allowing non-invasive in vivo detection of Aβ deposits [5, 6]. Amyloid PET contributes to a more accurate and earlier diagnosis of cognitively impaired patients with suspected AD, allowing the disease to be confirmed or excluded [4–6]. It also provides clinicians with information about the extent, location, follow-up changes, and response to anti-amyloid therapy effect on the Aβ deposition [1], and no contraindications have been described.

Currently, the use of amyloid PET imaging is considered clinically appropriate to estimate Aβ plaques in adult patients with cognitive impairment being evaluated for AD [2, 5].

#### Amyloid-Β-targeting tracers

The first amyloid PET radiotracer was the carbon-11 Pittsburgh Compound B ([^11^C]PiB), which is an analogue of Thioflavin T (fluorescent amyloid dye). [^11^C] PiB showed high affinity and high specificity for Aβ plaques, however, the 20-min short radioactive half-life of carbon-11 limits its use to centres with an on-site cyclotron [4, 6, 7].

[^11^C] PiB often serves as a reference standard to which other amyloid PET agents are compared [2].

For wider access and commercialisation of this technique, fluorine-18 labelled amyloid-β-targeting tracers were developed. Fluorine-18 [^18^F] has a longer radioactive half-life of 110 min which enables widespread distribution and use of these radiopharmaceuticals beyond the manufacturing site for routine clinical use [2, 6–8].

Three [^18^F] labelled compounds are approved for amyloid PET imaging by the U.S. Food and Drug Administration, European Medicines Agency, and other global regulatory agencies [2, 4, 5]: [^18^F]florbetapir (commercial name Amyvid^TM^; company Eli Lilly), [^18^F]florbetaben (Neuraceq^TM^; Life Molecular Imaging), and [^18^F]flutemetamol (Vizamyl^TM^; GE Healthcare). They are essentially equivalent in clinical practice, have high reproducibility across centres, and have low individual variability [1]. All three radiotracers have been neuropathologically validated showing a sensitivity of 92%, 98%, and 91%, with a specificity of 91%, 89%, and 100% for [^18^F]florbetapir, [^18^F]florbetaben, and [^18^F]flutemetamol, respectively [6–8].

All these tracers exhibit a high non-specific white matter uptake, which results in a distinctive white matter pattern in scans of healthy subjects. For example, Alzheimer’s patients with increased cortical amyloid plaques show a loss of the distinction between grey matter and white matter.

[^18^F]flutafuranol (formerly NAV4694) is another fluorinated compound also available and used in clinical trials but not currently approved for clinical use, which shows a similar brain distribution that [^11^C]PiB in terms of tissue contrast [2, 4, 5].

#### Imaging procedure and interpretation

No specific preparation of the patient or drug withdrawal is required for the amyloid PET scan [5]. Amyloid PET tracers differ in their tracing kinetics, specific binding ratios and optimal imaging parameters. Recommended injected activities, image acquisition times, and image interpretation details of the amyloid PET compounds are shown in Table 1 [2, 4, 5].

**Table 1:** Recommended injected activities, uptake phase, acquisition duration, recommended colour scale and image interpretation for the three FDA and EMA-approved [^18^F]amyloid-β-compounds

Radiotracer	Dose, (MBq)	Uptake phase, (min)	Acquisition, (min)	Colour scale	Reference region and normal SUVR, (NV)	N° regions for positive scan	Evaluated regions
[^18^F]florbetapir	370	30–50	10	Greyscale or inverse greyscale	Whole cerebellum NV ≤ 1.17	Two, or one if grey matter uptake exceeds white matter uptake	Temporal, parietal (including precuneus), frontal, and occipital
[^18^F]flutemetamol	185	90	20	Rainbow or Sokoloff	Pons NV ≤ 0.61	One	Temporal, parietal, posterior cingulate/precuneus, frontal, striatum
[^18^F]florbetaben	300	45–130	20	Greyscale or inverse greyscale	Cortical cerebellum NV ≤ 1.35	One	Temporal, parietal, posterior cingulate/precuneus, and frontal

**Fig. 1:**
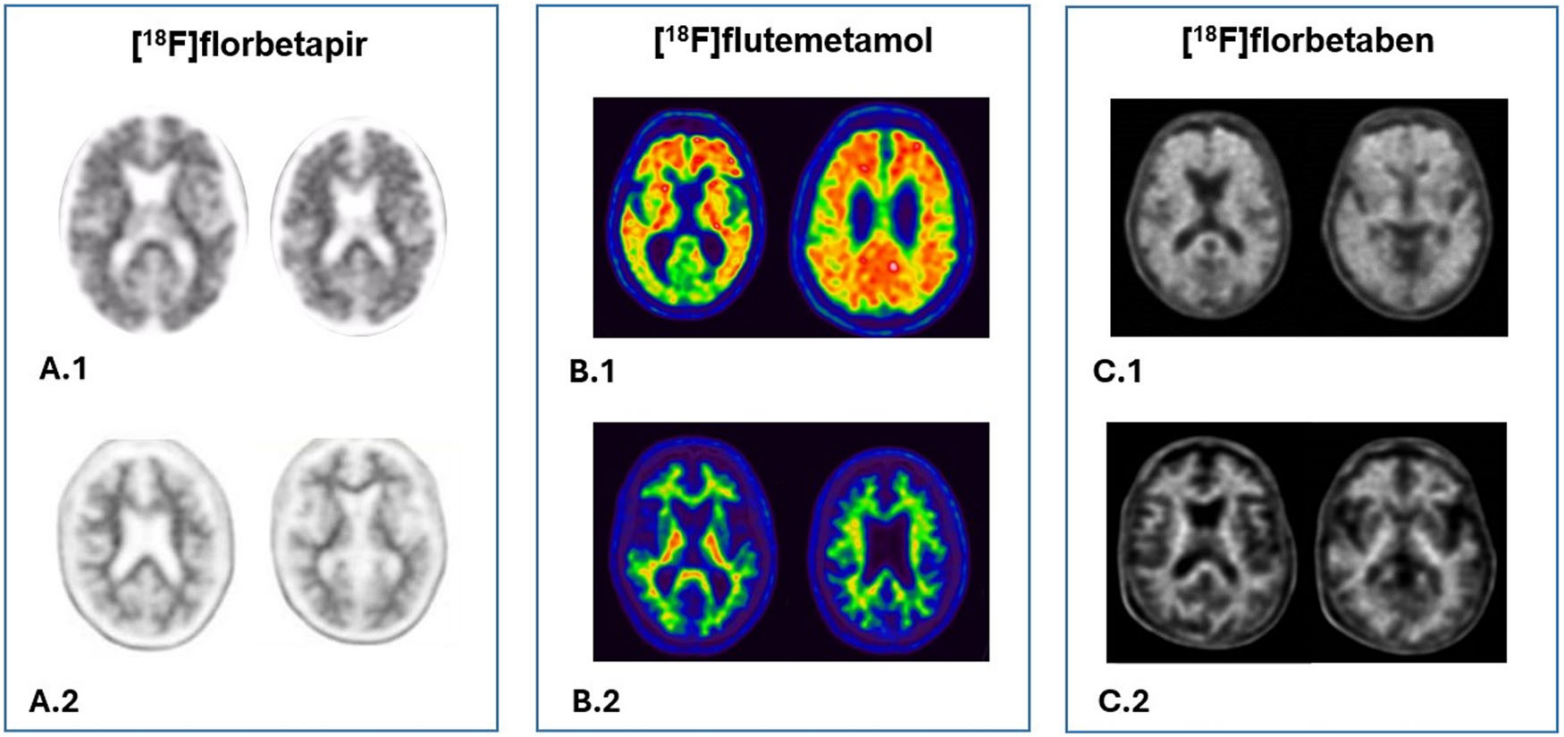
Axial PET images of [^18^F]florbetapir (**A**), [^18^F]flutemetamol (**B**), and [^18^F]florbetaben (**C**) in the colour scale recommended by the tracer manufacturer. Positive brain amyloid scans of Alzheimer’s patients in images 1 (A.1, B.1, and C.1) and negative brain amyloid scans in images 2 (A.2, B.2, C.2)

According to the insert package, PET images using [^18^F]florbetaben should be displayed in greyscale, with [18F]florbetapir in inverse greyscale, and with [^18^F]flutemetamol in Rainbow or Sokoloff colour scale. Additionally, image activity should be adjusted by setting the colour scale to different regions depending on the tracer employed [2, 5] (Fig. 1).

##### ***Visual reading***.

Interpretation of amyloid PET images is based on structured visual analysis, according to established recommendations for each of the three approved amyloid tracers. A qualitative binary interpretation as a positive or negative scan is performed. As a prerequisite, nuclear medicine physicians must complete an appropriate training programme provided by the radiotracer manufacturers [2, 5] (Table 1).

In general, the contrast between the high activity in the white matter and the uptake in the grey matter is evaluated. Negative amyloid PET scans show only non-specific tracer retention in white matter, resulting in a pattern resembling numerous concave arboreal ramifications that do not extend into the cortical ribbon (Fig. 1: A.2, B.2, and C.2). Besides, positive amyloid PET scans also show grey matter uptake, i.e. the grey-white junction is blurred due to tracer retention extending into the neocortex forming a smooth and regular boundary at the edge of the cerebral cortex [2, 5] (Fig. 1: A.1, B.1, and C.1).

All cerebral cortical and subcortical regions should be examined, with particular attention to the lateral temporal, frontal, posterior cingulate/precuneus, parietal cortices, and basal ganglia [4, 5]. Tracer uptake in the posterior cingulate, precuneus and frontal cingulate cortex is observed in the early stages, while generalised neocortical uptake is usually seen at later stages [2, 7].

In addition, the correlation of amyloid PET images fused with morphological images (MRI or CT) is recommended to assess sources of misinterpretation such as atrophy, vascular comorbidities, or structural abnormalities, among others [5]. The use of multimodality devices such as PET/CT or even PET/MR facilitates the fusion process, but the different imaging modalities can easily be fused using the software package currently available in the workstations Finally, it is important to keep in mind that a positive amyloid PET indicates the presence of high amyloid plaques (Alzheimer´s pathology), not the diagnosis of Alzheimer´s disease.

##### ***Quantification***.

Quantification of the amyloid burden by means of PET has been extensively used for inclusion criteria and monitoring the response to the anti-amyloid therapy effect of the Aβ deposits [7]. In clinical practice, it can complement visual analysis, particularly for inexperienced readers, when the confidence in visual analysis is low, or in borderline cases. Quantification of amyloid PET has been included as an adjunct to visual interpretation in European labels, but not in the FDA labels yet [2].

The most common quantitative measure is the standardised uptake value ratio (SUVr) which calculates the ratio between selected cerebral regions of interest (ROI) uptake (target regions that are known to accumulate amyloid plaques) and a reference region uptake (that is relatively spared of pathology) [2, 4, 6]. As ROIs vary depending on the radiotracer used, SUVr thresholds for a positive or negative scan differ from one [^18^F]-compound to another [6–8]. Interestingly, PET manufacturers are providing software to calculate automatically the SUVr of all three approved radiotracers.

The current trend is to use a single measure of amyloid burden, independent of the radiotracer used, that reflects the total pathological burden of amyloid. This will allow better comparison of data between centres and ultimately lead to the application of universal diagnostic and prognostic values.

This metric is named Centiloid, and the value is derived from the SUVr using a linear equation that transforms it into an unbounded scale from 0 to 100. The two parameters of this linear equation (a scale factor and an intercept) are specific to each tracer and pipeline analysis and should be calibrated with a test population to ensure that zero is associated with “high certainty” amyloid negative subjects and an average of 100 is associated with “typical” AD patients [9]. Once the calibration has been established, for example in commercial software or by a previous study, the calculation is straightforward.

In general, centiloid values below 10 excludes the presence of Aβ pathology, values above 40 centiloids correspond well with pathological amounts, while values falling in between these two cutoffs (“intermediate-range” or “grey-zone”) are related to an increased risk of disease progression [10]. Neuropathologic studies have shown that the earliest detectable amyloid PET signal occurs around 12 centiloids, the optimal threshold to predict future significant Aβ accumulation ranged from 15 to 17.5 centiloids in cognitively unimpaired participants, and 19 centiloids reflect the cut point beyond which the amyloid rate of change increases reliably beyond baseline variation in the measure. Centiloids between 21 and 24 best discriminate between subjects with no-to-low Aβ plaque burden and those with intermediate-to-high deposition, a cut-off value of 26 was found to best predict progression to dementia, and 30 centiloids are indicative of established Aβ burden [10, 11].

Furthermore, centiloid has been recently approved by the EMA to quantify brain amyloid deposition using amyloid PET as an adjunct to visual reading [12].

Centiloid quantification has been proposed as a valuable adjunct to the visual assessment of amyloid PET images to provide high confidence in identifying early or emerging Aβ pathology as intermediate values are associated with an increased risk of disease progression, to select patients for anti-amyloid disease-modifying therapies, and to measure the degree of Aβ clearance after treatment [10].

#### Pitfalls and artefacts

**Fig. 2:**
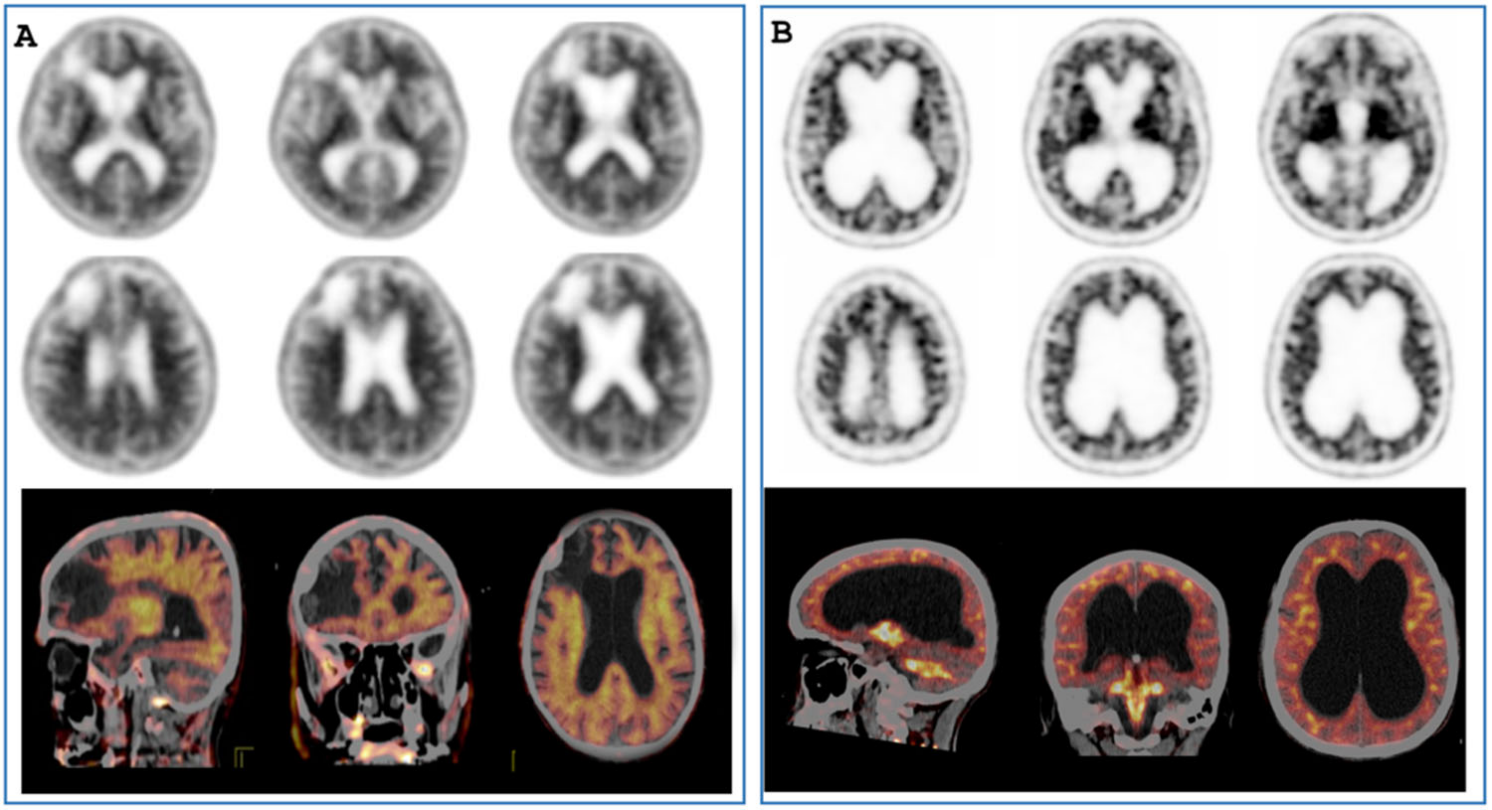
Pitfalls and artefacts in amyloid PET imaging. **A** [18F]Florbetapir PET/CT scan of a patient with right frontal stroke and brain atrophy. The PET/CT fusion images clearly show no difference between white matter and grey matter (positive amyloid PET scan). **B** [18F]Florbetapir PET/CT scan of a patient with normal pressure hydrocephalus. In this case, the fusion images showed much higher activity in the white matter than in the grey matter (negative amyloid PET scan)

Amyloid PET images should be correlated with a morphological imaging study to identify sources of error and provide useful information for those studies that pose a diagnostic challenge. Atrophy, which is common in ageing patients, complicates interpretation because it can lead to a false-negative scan. For example, in severe atrophy, it is difficult to distinguish a thin band of amyloid-positive grey cortex from non-specific uptake in the adjacent white matter. Moreover, it may lead to a false-positive scan due to overestimation of radiotracer uptake from the white matter uptake to the remaining cortex [4, 5]. Encephalomalacia from prior stroke, brain surgery, head trauma, and hydrocephalus might be some additional causes of misinterpretation [5] (Fig. 2). Finally, head movement during acquisition results in blurring of the image which decreases the diagnostic accuracy [4, 5]. Aβ plaques can be present in other non-AD dementias among which dementia with Lewy bodies has the highest amyloid positivity (51–70% of patients have diffuse Aβ plaque deposition), [2, 4, 5, 8], followed by vascular dementia (30%), frontotemporal dementia (12%), cerebral amyloid angiopathy, and which also increases with age and in APOE ε4 carriers. [2, 8, 13, 14]. In patients with corticobasal syndrome, the overall prevalence of Aβ plaques is 38% but does not increase with age; this is thought to be because AD may be the underlying pathology in young patients with corticobasal syndrome, whereas primary tauopathy becomes more likely with age [14].

### Tau PET imaging

Tau protein is mainly found in the axons of the central nervous system, where it plays a crucial role in stabilising microtubules. In the human brain, there are six isoforms of tau that are classified according to the number of the microtubule-binding domain repetitions, as three (3R) or four (4R) repeats [15, 16].

There are a number of neurodegenerative diseases called tauopathies that are characterised by the pathological accumulation of tau [15–17]. They are subdivided into primary tauopathies in which tau deposits predominate, and secondary tauopathies in which other protein aggregates predominate as in AD with Aβ deposits. In addition, tau protein deposits also vary in each of the neurodegenerative diseases, in terms of their morphology or ultrastructural conformations [16, 17] (Table 2).

**Table 2:** Histopathological appearance of tau isoforms and conformation in major tauopathies

	Tauopathy	Tau isoform	Ultrastructural conformation	Morphology
Primary tauopathies	PSP	4R	Straight filaments	Tufted astrocytes; globose tangles
	CBS	4R	Straight filaments mainly, and twisted filaments	Astrocytic plaques
	Pick’s disease	3R	Twisted filaments mainly, and straight filaments	Pick’s bodies
Secondary tauopathies	AD	3R and 4R	Paired helical filaments mainly, and straight filaments	Neurofibrillary tangles

*PSP* progressive supranuclear palsy, *CBD* corticobasal degeneration, *AD* Alzheimer’s disease

#### Tau PET tracers

The tau PET compounds initially developed were [^18^F]THK5317, [^18^F] THK5351, [^18^F]AV1451 (also known as [^18^F]flortaucipir), and [^11^C]PBB3) [2, 15]. However, [^18^F]flortaucipir was the first widely used tau agent, and was approved for clinical use by the FDA in 2020 and more recently by EMA in 2024. [18]. This first-generation of tau radiopharmaceutical showed to have a high affinity for paired helical filaments and neurofibrillary tangles for the different tau isoforms, which mainly characterise AD (AD-related tau) [15, 19]. Post-mortem studies have shown that tau PET radiopharmaceuticals bind selectively to phospho-tau and not to amyloid or other aggregates [15]. However, these tracers are limited by off-target binding (binding to intracerebral areas where tau aggregates are not localised) to the basal ganglia and the choroid plexus, brainstem (substantia nigra), and dural venous sinuses. Additionally, the [^18^F]THK compounds are shown to be displaced by monoamine oxidase (MAO) inhibitors [20].

These limitations prompted the development of a second-generation of tau tracers, including ^18^F]MK-6240, [^18^F]RO-948, [^18^F]RO1643, [^18^F]RO4693, [^18^F]PI-2620, [^18^F]GTP1, [^18^F]PM-PBB3, [^18^F]AM-PBB3, [^18^F]JNJ311, and [^18^F]JNJ-067, although none have yet received FDA or EMA approval [2, 15, 16]. These compounds were developed to minimise off-target binding, which significantly improves the assessment of tau accumulation, with a better ratio between pathological and normal values.

**Fig. 3:**
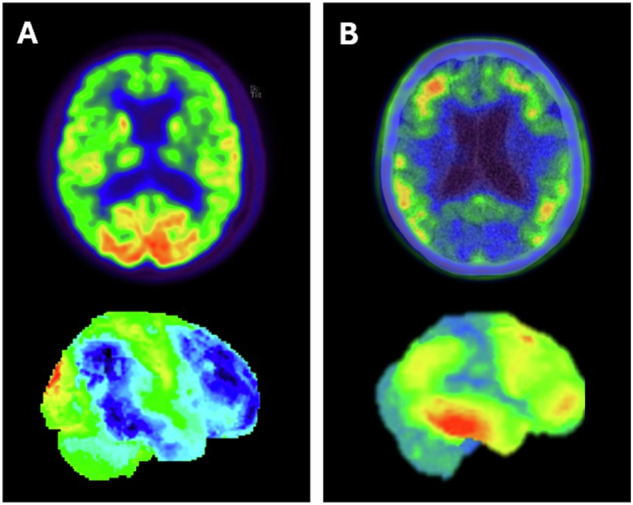
[^18^F]FDG PET (**A**) and [^18^F]flortaucipir (**B**) in an AD patient. Images show temporoparietal and frontal hypometabolism (**A**), which correlates with AD-related tau deposits

These new tracers have been shown in post-mortem studies to bind to AD-associated tau [21–23], and to correlate with AD dementia severity and symptoms, in contrast to Aβ [13, 16] (Fig. 3).

Some recently developed tau PET tracers, such as [18F]PI-2620, have also been shown to bind to 4R tauopathies, such as PSP or corticobasal degeneration (CBD), showing promising results for the diagnosis of other non-AD tau pathologies [15, 23, 24].

According to the FDA and EMA technical specification for [^18^F]flortaucipir, this compound is granted to estimate the density and distribution of aggregated tau NFTs for adult patients with cognitive impairment who are being evaluated for AD [18]. In addition, and as stated by the new appropriate use criteria, tau PET is particularly useful in MCI and dementia syndromes with atypical features or in subjects younger than 65 years, predict disease progression in MCI and determine eligibility for treatment with amyloid-targeting therapy [2]. As with amyloid PET, an “AD-like” tau PET binding pattern may help to establish AD as the primary or contributing cause of impairment. In addition, the spatial pattern of tau PET often matches brain regions that are clinically affected and show evidence of neurodegeneration on FDG-PET or MRI [25–28] increasing the confidence that the underlying syndrome is due to AD. Similarly, tau PET can be very helpful in detecting AD pathology in young-onset AD, with higher overall intensity and spatial distribution of radiotracer retention compared with older patients at a similar disease stage [29]. Because early-onset AD patients are more likely to have advanced Braak stages of neurofibrillary pathology even at the MCI stage [29] this increases the likelihood of a positive tau PET scan.

In addition, high tau burden is associated with more rapid clinical progression and cognitive decline in subjects with MCI, which is associated with tau protein deposition, and has been shown to correlate more closely with dementia severity than amyloid burden [30].

The use of tau PET in clinical trials of disease-modifying treatments is relatively limited. However, the data available to date suggest that baseline tau PET may predict the magnitude of clinical benefit associated with amyloid clearance by monoclonal antibodies as it appears that amyloid clearance appears to be more beneficial in individuals with earlier stages of tau progression according to PET. In the TRAILBLAZER-ALZ2 study [31] the “low-medium” tau PET group showed a greater slowing of clinical decline and the “combined population”, which includes participants with high tau PET showed limited clinical benefit compared to placebo. Similarly, in the CLARITY-AD sub-study analysis of tau PET patients with the lowest baseline tau PET showed the greatest clinical benefit from treatment [32].

#### Imaging procedure and interpretation

No special patient preparation or drug withdrawal is required for tau PET imaging [16]. For [^18^F]flortaucipir PET images are obtained 80–100 min after the injection of 370 Mbq [2, 16]. Images should be displayed in axial, coronal, and sagittal views using a colour scale with a rapid transition between two different colours, adjusting the scale so that the transition occurs at the 1.65-fold threshold [2], or until the striatum appears with the appropriate background, taking the cerebellar cortex as a predefined reference background (negative control) [16]. It is necessary to evaluate the posterolateral temporal region first, followed by the occipital, parietal and frontal regions bilaterally.

To assess the temporal lobes, they are divided into four quadrants in the axial view by placing the horizontal crosshairs immediately posterior to the brainstem nuclei and then scrolling inferiorly to place the vertical crosshairs through the widest part of the temporal pole, thus obtaining the anterolateral temporal, anterior mesial temporal, posterolateral temporal, and posterior mesial temporal quadrants [33].

#### Visual reading

A positive AD tau pattern shows neocortical uptake in posterolateral temporal, occipital, or parietal/precuneus regions in both hemispheres, with or without frontal activity in more advanced disease (according to Braak and Braak stages) [33, 34] On the other hand, a negative AD tau pattern is defined when there are no neocortical tau deposits but normal uptake in the midbrain, caudate, putamen, pallidum and thalamus is present [2, 16] or with increased neocortical uptake isolated to the mesial temporal, anterolateral temporal, and/or frontal regions [33].

In the visual analysis is recommended the co-registration of [^18^F]flortaucipir PET with anatomical imaging (such as CT or MRI) for definition and localisation of tau deposits [16].

#### Quantification

Tau burden can be quantified by calculating the SUVr a ratio between the volumes of interest (VOIs) of the Braak stage regions and the SUVs of the inferior cortical cerebellum (reference region) [16]. Quantification of tau deposits in AD can increase the sensitivity of early stages, assist with disease staging and measure the longitudinal change in tau burden as a result of disease progression or in response to therapeutic interventions [2].

Recently the CenTauR (CTR) scale has been proposed, designed to standardise the quantification of tau in PET images of different tau-PET tracers for AD, with values anchored between cognitively normal individuals and patients with Alzheimer’s dementia, similar to the Centiloid scale. CTR provides a unified measure of tau burden and improves data interpretation for research and clinical trials [35].

### Pitfalls and artefacts

[^18^F]flortaucipir PET images need to be evaluated prior to interpretation to detect possible off-target binding of [18F]flortaucipir in the choroid plexus, brainstem, basal ganglia and meninges (Fig. S1). As well as to detect other sources of error such as the existence of patient’s head movement, unexpected focal lesions or cortical atrophy [16].

## Neuronal activity and neurodegeneration: [^18^f]FDG PET

Two distinct molecular imaging techniques may be employed to evaluate neuronal activity and neuronal degeneration, namely PET using [^18^F]FDG, and perfusion single-photon emission computed tomography (SPECT) using technetium-99m hexamethylpropyleneamine oxime ([^99m^Tc]HMPAO). Approximately 95% of the energy required for brain function is derived from glucose metabolism, which is closely linked to neuronal activity under physiological conditions. [^18^F]FDG, is a glucose analogue that is transported into the brain via glucose transporters, and metabolised by hexokinase to glucose-6-phosphate [1, 3]. Specifically in the brain, [^18^F]FDG is transported from the capillaries to the astrocytic end-feet which are enriched in GLUT3 (activated by glutamate). [^18^F]FDG is metabolised into lactate, which subsequently serves as an energy source for neurons (“astrocyte-neuron lactate shuttle”) predominantly to glutamatergic excitatory synapses that consume a large portion of the [^18^F]FDG uptake in the human brain [36]. However, the measurement of cerebral blood flow by means of perfusion SPECT provides an indirect assessment of neuronal activity through the analysis of blood perfusion patterns [37]. In recent years, there has been a growing preference for the use of [^18^F]FDG PET imaging over perfusion SPECT imaging in certain clinical contexts, particularly due to its superior diagnostic accuracy and spatial resolution.

In neurodegenerative conditions with cognitive impairment, specifically in AD, [^18^F]FDG PET is viewed as a marker of neurodegeneration (N) and progression, and currently included—along with hippocampal volume measured with MRI—in the A/T/N classification scheme with amyloid-ß (A) and tau (T). [^18^F]FDG PET is recommended to support early diagnosis of AD in MCI (predict AD conversion), frontotemporal lobar degeneration and dementia with Lewy bodies (in addition to presynaptic dopaminergic imaging which usually is more accurate in this indication) [38]. A recent consensus algorithm has been proposed on suitable indications of [^18^F]FDG PET, especially emphasising its great value as a first-line evaluation when a non-AD disorder is clinically suspected. The recognition of the transactive response DNA binding protein of 43 kDa (TDP-43) proteinopathy (LATE) on [^18^F]FDG PET is particularly important as there is currently no imaging biomarker of TDP-43, and also AD and LATE pathologies overlap significantly in elderly patients [3]. Additionally, [^18^F]FDG PET can be used for the differential diagnosis between Parkinson´s disease (PD) and atypical parkinsonian syndromes such as PSP, multiple system atrophy (MSA), corticobasal syndrome (CBS), and the already mentioned dementia with Lewy bodies [1, 39].

### Procedure and interpretation

Patient pre-arrival preparation consists of a fasting period of at least 4–6 h, and parenteral feeding or intravenous fluid therapy with dextrose should be withdrawn for 4–6 h [39–41]. Prior to [^18^F]FDG injection, blood glucose levels should be checked to exclude hyperglycaemia (>110 mg/dL) [39–41].

After the injection of [^18^F]FDG (usually 125–250 MBq in adults), patients must rest comfortably in a dimly lit and quiet room for 30–40 min before scanning to minimise variation of uptake during tracer accumulation. To avoid sensory and motor stimulation of the patient, they should be instructed not to speak, read, listen to music/sounds, and be awake with their eyes open [39–41].

#### Visual reading

[^18^F]FDG brain PET images are presented in a colour scale with a continuous progression of the colours from low to high uptake because it allows for better recognition of the areas with reduced metabolism, whereas the grey scale only allows for easily identifying hot areas [40–42]. Spectrum or rainbow colour scales are commonly used, and they should be adjusted to show basal ganglia in red colour, white matter in green-red, and cerebrospinal fluid as a deep blue part of the spectrum [41]. A standardised image display with proper orientation is a prerequisite to ensure appropriate and reproducible reading [41].

#### Quantification

**Fig. 4:**
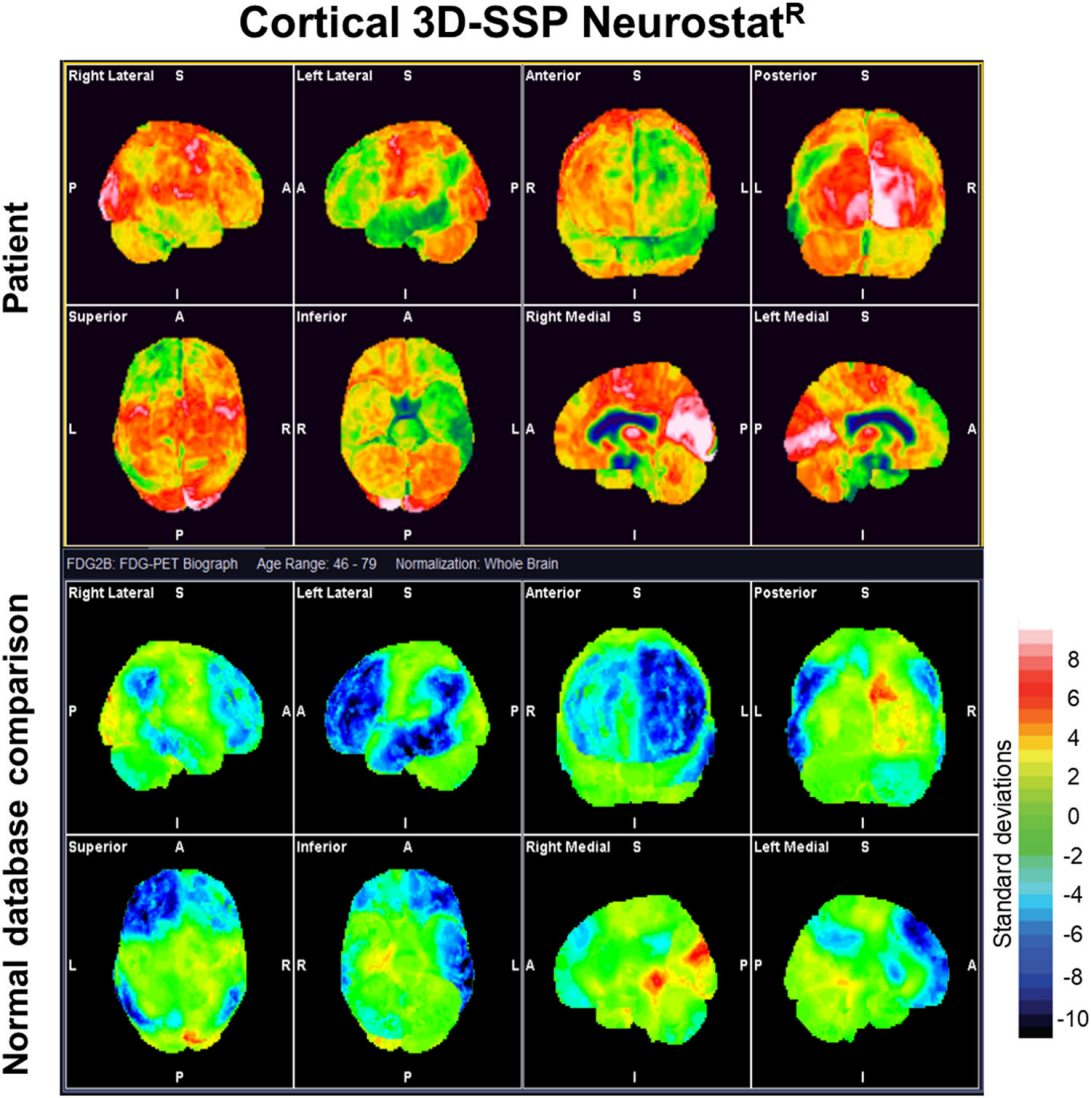
Stereotactic surface projection displaying local deviations of individual patients from an age-adjusted normal reference sample (3D surface z-maps)

Automated scoring and semi-quantification tools are currently available from PETscanner manufacturers and other vendors, improving the diagnostic performance of readers in clinical settings. Tools for semi-quantification and voxel analysis provide individual statistical maps (parametric or *z*-score maps) aimed to support visual reading and improve anatomical localisation of regions of abnormal metabolism. It improves diagnostic performance, especially of moderately skilled readers, but it should always be used in conjunction with visual reading (considering always the first step) [39, 40]. It provides spatial normalisation and mapping of local deviations of individual patients from a normal reference sample adjusted for age (3D surface z-maps) [39, 41]. Scaling (intensity normalisation) of the regional [^18^F]FDG uptake is required for this purpose. This is done by normalisation to the whole brain or to predefined reference regions known to be unaffected in specific clinical contexts, such as the pons [39, 40]. Various freeware and commercial software are available, such as 3D-SSP (Neurostat^R^). This tool has been specifically designed for single-subject analysis and includes a group of normal control databases. It provides a stereotactic surface projection display using different reference regions for normalisation (whole brain, pons, thalami and cerebellum), allowing the user to appreciate the effect of the different reference regions on the final results. [39, 40] (Fig. 4).

### Pitfalls and artefacts

The presence of patient movement during PET acquisition or between PET and CT or MRI should be considered as it can produce blurred images and attenuation artefacts [39, 40]. Morphological changes like atrophy, cortical sulci, and ventricle enlargement are frequent causes of hypometabolism and should be identified.

It is also important to register patient treatment as some drug interference in the CNS can reduce thalamus and cortex metabolism. Moreover, high glucose levels could reduce grey matter contrast and mimic some patterns of neurodegenerative conditions.

**Fig. 5:**
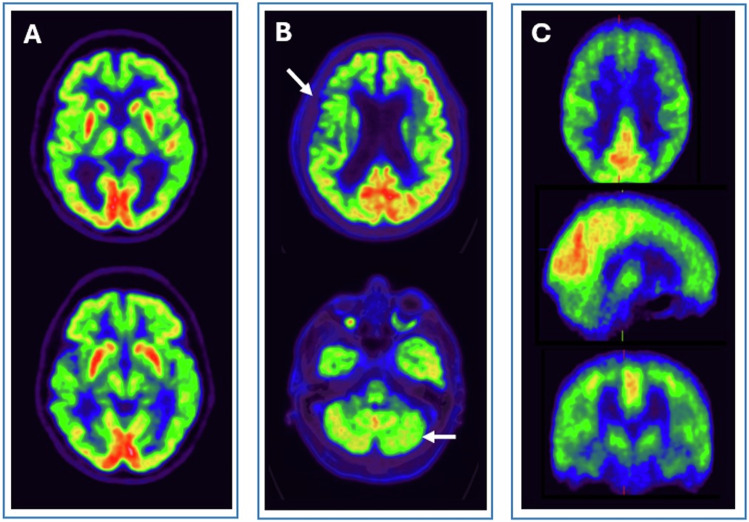
Pitfalls and artefacts in [^18^F]FDG PET imaging. **A** Scan of a patient with CNS drug interference showing cortical hypometabolism that could mimic an AD pattern, but with thalamic hypometabolism characteristic of this interference. **B** Scan of a patient with a right frontoparietal ischaemic antecedent (arrow), showing a diaschisis of the contralateral cerebellum (arrow) due to disruption of the corticospinal tract. **C** An example of a PET scan with motion artefacts

Finally, the effect of deafferentation or diaschisis should be taken into account when focal vascular o neurodegeneration is present [3] (Fig. 5).

## Dopaminergic neurotransmitter imaging

Dopaminergic neurons reside predominantly in the midbrain, and project predominantly to the striatum via the nigrostriatal pathway [19, 43].

Dopamine (DA) is synthesised in presynaptic dopaminergic neurons and transported to intracellular vesicles by vesicular monoamine transporter 2 (VMAT_2_), to be stored and protected from oxidation by MAO. Presynaptic dopaminergic neurons release DA into the synaptic cleft in response to an action potential, interacting DA with postsynaptic DA receptors (D_1_/D_2_). To terminate this interaction, DA is reuptake into the presynaptic dopaminergic neuron by the dopamine transporter (DaT) [42, 43] (Fig. S2).

### Dopaminergic targeting tracers

Although this review focuses on molecular PET imaging, in the case of presynaptic dopaminergic imaging, SPECT imaging using radiotracers that bind the striatal DaT must be mentioned, as they are currently the most widely used in clinical practice. This group of radiotracers reflects DaT density and correlates with loss of nigrostriatal DA terminals and includes SPECT radionuclides such as [^123^I]FP-CIT ([^123^I]-ioflupane), [^123^I]β-CIT, [^99m^Tc]TRODAT, and more recently PET radiotracers such as [^18^F]FE-PE2I [19, 42, 43]. Additionally, PET using L-6-[^18^F]fluorodopa ([18 F]FDOPA) reflects the structural and the biochemical integrity of the presynaptic dopaminergic nerve terminals as it is metabolised to [^18^F]DA and stored in synaptic vesicles [19, 42, 43]. Other tracers that reflect the integrity of the presynaptic dopaminergic terminal are those that bind to vesicular monoamine transporter type 2 (VMAT_2_), such as tracers derived from tetrabenazines ([^11^C]DTBZ, [^18^F]DTBZ) [19]. Nevertheless, only [^123^I]FP-CIT and the [^18^F]FDOPA are currently approved by the FDA and EMA for clinical purposes [1, 42].

At the postsynaptic level, tracers that bind to the postsynaptic DA receptors reflect the integrity of the postsynaptic dopaminergic neurons. The 90% of the postsynaptic DA receptors are D_2_ type. Among these tracers are [^18^F]fallypride, [^18^F]desmethoxyfallypride, [^11^C]raclopride [42–44]. However, postsynaptic dopaminergic imaging is currently not clinically available.

### Presynaptic dopaminergic imaging

Presynaptic dopaminergic imaging is indicated for detecting the loss of nigrostriatal dopaminergic neuron terminals of patients with parkinsonian syndromes. It supports the differential diagnosis between neurodegenerative causes of parkinsonian syndromes (dopaminergic deficit) and non-DA deficiency aetiologies of parkinsonism where studies shown the integrity of the nigrostriatal dopaminergic neurons (essential tremor, drug-induced, psychogenic, or vascular parkinsonism) [2, 42, 43]. However, presynaptic dopaminergic imaging is unable to distinguish between the different neurodegenerative parkinsonian syndromes. [45]. Finally, it helps the differentiation of dementia with Lewy bodies from other dementias with no nigrostriatal dopaminergic neuron loss (particularly AD) [2, 42].

### Procedure and interpretation

Patient preparation, injection, uptake phase and acquisition protocol for [^123^I]FP-CIT and [^18^F]FDOPA are summarised in Table 3.

**Table 3:** Patient preparation, injection, uptake phase and acquisition protocol

		SPECT using [^123^I]FP-CIT	PET using [^18^F]fluorodopa
Patient preparation	Dietary restrictions	None	Amino acid-containing foods 4 h prior to avoid a competition in transfer by a common carrier through the blood-brain barrier
	Drug withdrawal	Should stop for at least 5 half-lives drugs that bind with high affinity to DaT that may decrease striatal [123I]FP-CIT binding: -Cocaine (2 days) -Amphetamines (7 days) -CNS stimulants: phentermine or ephedrines (1 day) -Modafinil (3 days) -Antidepressants: mazindol, bupropion, radafaxine (3 days) -Adrenergic agonists: Phenylephrine or norepinephrine -Opioids: Fentanyl (5 days) -Anaesthetics: Ketamine, phencyclidine, isoflurane (1 day) -Anticholinergic drugs: Benztropine	Anti-parkinsonian drugs (LDOPA) at least 12 h improve image quality (although it should not interfere with visual interpretation significantly)
Injection	Preinjection pretreatment	Potassium iodide oral solution/Lugol’s solution (equivalent to 100 mg iodide) or potassium iodide tablet, potassium perchlorate (400 mg), or sodium perchlorate (600 mg) at least 1 h prior, to reduce radiation exposure to the thyroid	Carbidopa (150 mg or 2 mg/kg up to 150 mg, orally) or Entacapone (200 mg, orally) 60–90 min prior, increases the availability of DOPA to the brain and reduces absorbed dose to the bladder and kidney
	Administered dosage	110–250 MBq (typically 185 MBq) in adults	185 Mbq in adults
	Uptake phase	3–6 h	70–90 min
Acquisition protocol		25–40 s per frame	10–20 min static image

Iodine allergy is not a contraindication to [^123^I]FP-CIT but it is a contraindication for iodine-containing pretreatment [42]

Images should be read on a colour scale, and the striatal activity level should be compared with the background activity [42]. Furthermore, available morphological information (existing CT or MRI scans) should be checked for morphological changes in the basal ganglia or in the selected reference region [42]. The reorientation procedure must be reformatted in three orthogonal planes for visual assessment. Axial sections parallel to a given anatomical orientation ensure a high degree of standardisation in-plane orientation to avoid artefactual asymmetry that may lead to visual misinterpretation in the generation of coronal and sagittal sections [42].

### Visual reading

Normal [^123^I]FP-CIT SPECT scans show symmetrical with well-delineated striatal borders, as a “comma-shaped” [42]. In abnormal scans, the striatal looks like a “dot shape” due to the more severely affected putamen (in particular the posterior part) than the caudate nucleus. In PD patients striatal uptake is first reduced in the putamen contralateral to the side of the body showing the most severe signs of disease, then the reduction in tracer uptake extends over time to the contralateral putamen and anteriorly and ipsilaterally to the caudate [42, 45]. Other neurodegenerative parkinsonian syndromes (PSP, MSA…) compared to PD can exhibit a prominent uptake reduction in the caudate nucleus, which may result in striatal images with “weak commas” or “balanced loss”. However, it is important to remember that these abnormal patterns of presynaptic dopaminergic impairment do not allow reliable differentiation between the various neurodegenerative forms of Parkinsonism [42].

**Fig. 6:**
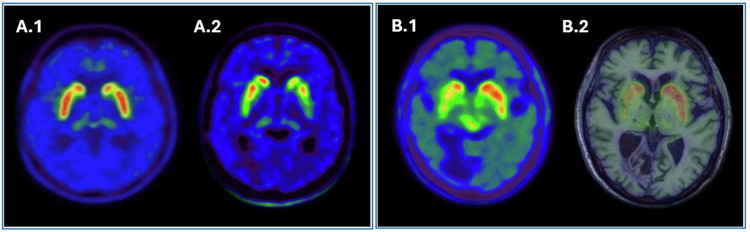
**A** [^18^F]FDOPA PET image of a healthy patient (A.1) and a patient with PD (A.2). **B** Pitfalls and artefacts in presynaptic dopaminergic imaging. [^18^F]FDOPA PET scan (B.1) of a patient with suspected Parkinson’s disease showing a decrease in the right putamen. However, the fusion PET/MRI image (B.2) shows that the decreases are due to the artefact of spaces surrounding the walls of vessels within the brain parenchyma observed on MRI

PET images provide higher spatial and temporal resolution than SPECT, which facilitates the evaluation of different parts of the striatum making possibly easier the interpretation [42, 45]. In addition, [^18^F]fluorodopa striatal uptake is much less dependent on age than DaT tracers [42] (Fig. 6 A).

#### Quantification

Semiquantification analysis can be a complement to visual interpretation and improve diagnostic performance. The most common parameter used in [^123^I]FP-CIT SPECT and [^18^F]FDOPA PET images is the striatal target-to-background ratio (SBR). The reference region used to assess non-specific binding should have an absent or low dopaminergic terminal density, such as the occipital cortex and the cerebellum, and the SBR values are obtained as follows: (Mean counts of striatal VOI − Mean counts of background VOI)/(Mean counts of the background VOI) [42].

There are several commercial and free software programmes that perform automated semiquantification. It performs automatic SPECT or PET registration and spatial normalisation, place predefined VOIs in striatal and background regions, and provides SBR, left/right putamen/caudate asymmetry parameters, putamen/caudate ratio and caudate-putamen ratio. [42]. The results can be compared with a normal subject database that gives us the reference range and/or *z*-score differences of the patient studied with respect to the database, where *z*-score values < −2 are considered to be pathological [42].

### Pitfalls and artefacts

When assessing the images obtained from dopaminergic studies, we must confirm that there is no interference from medications or tracer extravasations, which may lead to a decrease in signal-to-noise ratio in patients with very mild motor symptoms. Ageing is associated with a 5.5–6% signal loss per decade (0.6%/year) on DaT tracers [42].

Moreover, dopaminergic images should be correlated with morphological imaging to rule out any structural lesions in the basal ganglia and midbrain. Vascular lesions often give a “punched-out” appearance, particularly in high-resolution images [42, 45]. Moreover, enlargement of perivascular spaces (Virchow–Robin spaces) may lead to a reduction in striatal uptake following the distribution of spaces [42] (Fig. 6 B).

## Conclusions

Different molecular imaging approaches are currently available to help the clinical diagnosis of neurodegenerative diseases with well-defined procedure guidelines. Existing PET imaging of pathological aggregates like Aβ plaques and tau neurofibrillary tangles using fluorinated radiotracers are particularly useful in clinical practice for the assessment of patients with cognitive impairment. The consistent standardised visual reading of amyloid PET images results in a low inter- and intra-subject variability, which is enriched by quantitative parameters. Specifically, the Centiloid scale is particularly useful to determine eligibility and response assessment to recent anti-amyloid targeting therapies. Tau PET imaging is an AD biomarker that provides accurate diagnostic information with regional correlation with clinical phenotype (differential diagnosis), and prognostic information of conversion from SCD and MCI to AD dementia. In the future, tau PET imaing could serve as an universal biomarker tool providing information on the A, T, and N status. Evaluation of neuronal activity and neurodegeneration by means of [^18^F]FDG PET, a worldwide highly available technique included in the majority of clinical diagnostic criteria of neurodegenerative diseases, requires proper patient preparation, as well as PET facility and personnel training. Presynaptic dopaminergic imaging has been used for years in the clinical diagnosis of neurodegenerative conditions with parkinsonism, but the recent development of new DaT PET tracers and the implementation of quantification for optimal reporting are improving its clinical value.

## Supplementary information

ELECTRONIC SUPPLEMENTARY MATERIAL

## Abbreviations

AD: Alzheimer’s disease
[^11^C]PiB: Carbon-11 Pittsburgh Compound B
CTR: CenTauR scale
CBD: Corticobasal degeneration
DA: Dopamine
DaT: Dopamine transporter
MAO: Monoamine oxidase
PET: Positron emission tomography
PSP: Progressive supranuclear palsy
SPECT: Single-photon emission computed tomography
SUVr: Standardised uptake value ratio
VMAT_2_: Vesicular monoamine transporter 2
Aβ: β-Amyloid
