# Supplementary material for: The basics of PET molecular imaging in neurodegenerative disorders with dementia and/or parkinsonism
Source: Eur Radiol. 2025 Feb 6;35(8):4621–34. doi: 10.1007/s00330-025-11388-5 (PMC12226624; doi:10.1007/s00330-025-11388-5)
Supplement: Supplementary file 1 — ELECTRONIC SUPPLEMENTARY MATERIAL [file 330_2025_11388_MOESM1_ESM.pdf]

The basics of PET molecular imaging in neurodegenerative disorders with dementia and/or parkinsonism.

ELECTRONIC SUPPLEMENTARY MATERIAL

FIGURE S1. Pitfalls and artefacts in tau PET imaging Transversal, coronal and sagittal slices of an [18F] flortaucipir PET/CT scan of an 82-year-old female healthy control demonstrate the presence of off-target binding in the basal ganglia and meninges, as well as the absence of neocortical tau deposits.

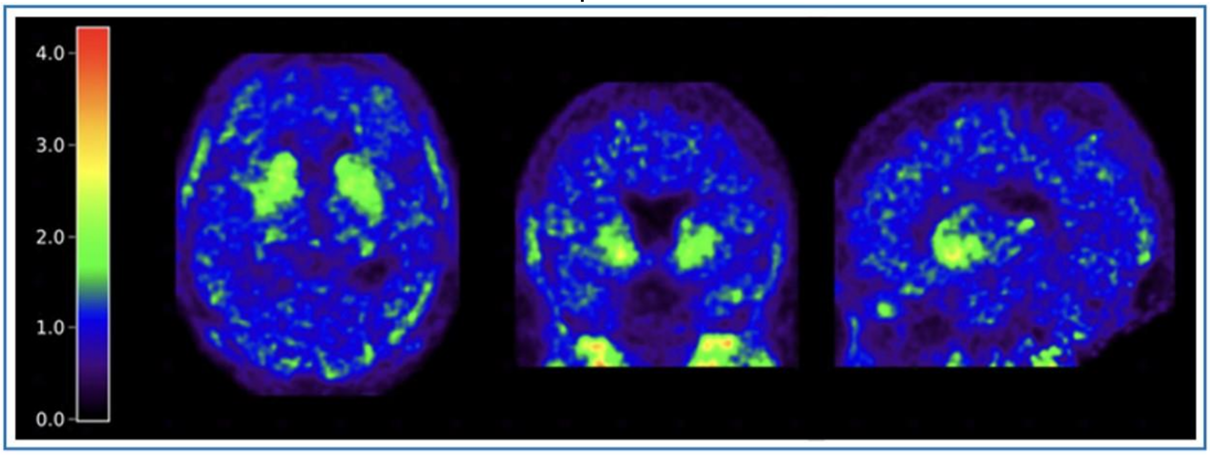

The image is courtesy of Dr Alexander Drzezga and Merle Hönig, Department of Nuclear Medicine, University Hospital of Cologne (Germany).

FIGURE S2. Dopaminergic nerve terminal and radiotracers for dopaminergic imaging.

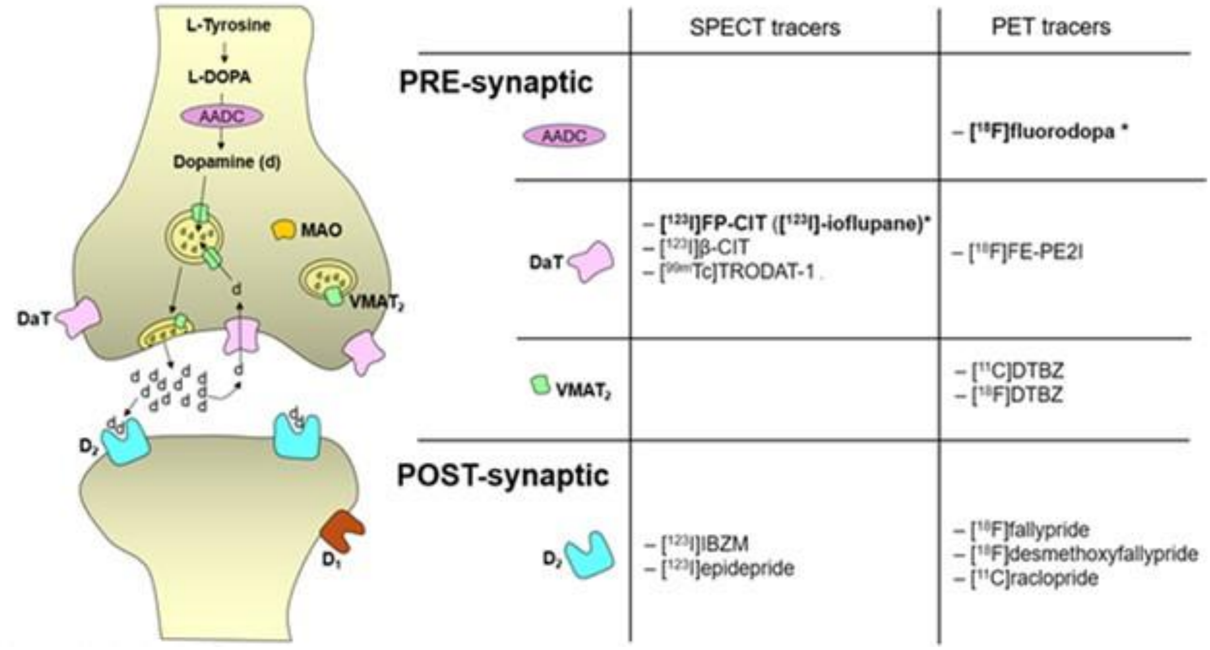

	SPECT tracers	PET tracers
**PRE-synaptic**		
AADC		**– $[^{18}F]$fluorodopa ***
DaT	**– $[^{123}I]$FP-CIT ($[^{123}I]$-ioflupane)*** – $[^{123}I]$β-CIT – $[^{99m}Tc]$TRODAT-1	– $[^{18}F]$FE-PE2I
VMAT$_2$		– $[^{11}C]$DTBZ – $[^{18}F]$DTBZ
**POST-synaptic**		
D$_2$	– $[^{123}I]$IBZM – $[^{123}I]$epidepride	– $[^{18}F]$fallypride – $[^{18}F]$desmethoxyfallypride – $[^{11}C]$raclopride

*FDA & EMA approved
